# Impact of Integration of FO Membranes into a Granular Biomass AnMBR for Water Reuse

**DOI:** 10.3390/membranes13030265

**Published:** 2023-02-23

**Authors:** Pere Olives, Lucie Sanchez, Geoffroy Lesage, Marc Héran, Ignasi Rodriguez-Roda, Gaetan Blandin

**Affiliations:** 1 LEQUIA, Institute of the Environment, University of Girona, 17003 Girona, Spainmailto:; 2 Institut Européen des Membranes (IEM), Université de Montpellier, CNRS, ENSCM, 34090 Montpellier, France

**Keywords:** anaerobic membrane bioreactor, granular biomass, membrane fouling, forward osmosis

## Abstract

The granular sludge based anaerobic membrane bioreactor (G-AnMBR) has gained emphasis in the last decade by combining AnMBR advantages (high quality permeate and biogas production towards energy positive treatment) and benefits of granular biomass (boosted biological activity and reduced membrane fouling). With the aim to further reduce energy costs, produce higher quality effluent for water reuse applications and improve system efficiency, a forward osmosis (FO) system was integrated into a 17 L G-AnMBR pilot. Plate and frame microfiltration modules were step by step replaced by submerged FO ones, synthetic wastewater was used as feed (chemical oxygen demand (COD) content 500 mg/L), with hydraulic retention time of 10 h and operated at 25 °C. The system was fed with granular biomass and after the acclimation period, operated neither with gas sparging nor relaxation at around 5 L.m^−2^.h^−1^ permeation flux during at least 10 days for each tested configuration. Process stability, impact of salinity on biomass, the produced water quality and organic matter removal efficiency were assessed and compared for the system working with 100% microfiltration (MF), 70% MF/30% FO, 50% MF/50% FO and 10% MF/90% FO, respectively. Increasing the FO share in the reactor led to salinity increase and to enhanced fouling propensity probably due to salinity shock on the active biomass, releasing extracellular polymeric substances (EPS) in the mixed liquor. However, above 90% COD degradation was observed for all configurations with a remaining COD content below 50 mg/L and below the detection limit for MF and FO permeates, respectively. FO membranes also proved to be less prone to fouling in comparison with MF ones. Complete salt mass balance demonstrated that major salinity increase in the reactor was due to reverse salt passage from the draw solution but also that salts from the feed solution could migrate to the draw solution. While FO membranes allow for full rejection and very high permeate purity, operation of G-AnMBR with FO membranes only is not recommended since MF presence acts as a purge and allows for reactor salinity stabilization.

## 1. Introduction

Water resources availability is being affected by scarcity, pollution, or access limitation [1]. For this reason, it is urgent to find alternative water sources, such as wastewater reuse. However, technologies need to be highly efficient, resilient, and reliable [2], which can be accomplished by improving existing technologies, such as membrane bioreactors (MBR). MBR integrate selective membranes within biological reactors and were developed during the 1960s and 1970s [3]. Membranes used in MBR are porous membranes (i.e., microfiltration (MF) or ultrafiltration (UF)), which allow the rejection of suspended solids, macromolecules such as proteins and some pathogens, but are not efficient enough to reject smaller molecules such as salts, pesticides, or pharmaceuticals, which are of high concern in the context of water reuse [2].

During the 1980s the anaerobic membrane bioreactors (AnMBR) were developed with the objective of recovering useful resources from wastewater, transforming organic matter into biogas, apart from the elimination of other pollutants [4]. The anaerobic digestion offers additional advantages over aerobic digestion thanks to its lack of aeration and its associated costs; it also produces less residual sludge which reduces disposal costs. Membrane fouling mitigation is a crucial aspect; air sparging is typically used in (aerated) MBR while biogas is used as gas sparging in some AnMBR configurations to reduce fouling effects on membranes [5]. Still, fouling remains a major hindrance in the scale-up of AnMBR together with the necessity to work under mesophilic conditions which negatively impacts the energy balance of the system.

To date, AnMBR is mostly implemented in high organic load industrial streams; operation in urban wastewater (WW) remains more challenging due to the low organic load. In the last few years, many studies focused on the direct treatment of municipal WW via AnMBR at lab and pilot-scale [6]. However, its broader development is still limited since the low methane production hardly offset the energy demand from membrane operation (biogas sparging and permeate pump) [7]. Recently, granular biomass based AnMBR (G-AnMBR) has gained interest since granules boost biomass activity, increase microbial diversity, improve resistance to shocks and reduce fouling [8]. It is hypothesized that the large size and solid structure of granular biomass combined with immobilization of extracellular polymeric substances (EPS) within the granule structure limit fouling, i.e., pore blocking, deposition and thickness of the cake layer on the membrane surface compared to conventional AnMBR [9,10]. Moreover, a very recent study proved that G-AnMBR applied for domestic wastewater at psychrophilic temperatures could achieve high organic matter removal rates, increasing effluent quality, while producing a net energy balance due to the biogas production, derived from the organic matter conversion to methane [11]. Such a configuration brings more opportunities for implementation of AnMBR in urban wastewater treatment schemes.

In parallel, forward osmosis (FO) gained some interest since it relies on the osmotic gradient, using dense membranes and is demonstrated to have a lower fouling propensity. Unlike MF and UF membranes, FO retains salts, pesticides, pharmaceutical compounds [12,13]. Combining FO with AnMBR has been used to increase COD load and improve biogas production. These technologies can be combined in two different ways: (1) by replacing or coupling the MF or UF membrane system with a FO system in an Anaerobic Osmotic MBR (AnOMBR) system or (2) by using FO to pre-concentrate WW for subsequent anaerobic treatment. Operation of AnOMBR positively led to almost total COD removal. Operating AnMBR only with FO membrane led, however, to high rejection rates, moderate fouling and severe salinity build-up over time when only an FO membrane is used [14]. High salinity has been found to be an important limiting factor of the AnMBR system due to its inhibitory or toxic effects on active biomass [15]. Tang et al. observed that it negatively affected methanogenic growth leading to ousting of methanogens by sulfate reducing bacteria [16]. Still, if the salinity shock also observed into MBR led to a rapid decrease of process efficiency, full recovery was observed after several days of operation demonstrating the potential of bacteria to overcome changes in salinity after an acclimation period [17]. Other studies demonstrated that, following salinity increase in an MBR, halophobic bacteria were replaced by halophilic ones leading to a proper operation even at high salinity [18]. Chen et al. also reported that operation at higher salinity did not impact the long-term production of biogas [19]. Still, the salinity issue is of potential concern. Combining FO and MF membrane into the AnOMBR reactor avoided severe salinity build-up while assuring production of high water quality (through the FO membrane), production of biogas and concentration of nutrients (phosphorous in the MF permeate) to facilitate its downstream recovery or reuse [20]. Still, the impact of salinity build-up when FO membranes are coupled to an AnOMBR reactor remains a potential limitation to be further studied. Salinity increase in AnOMBR is the consequence of feed concentration and passage of some salts from the draw solution (in the opposite direction of water) which could end in the reactor increasing the salinity rate. Salinity increase in the bioreactor could lead to cellular plasmolysis, increasing the fouling effects, or even could cause biomass death.

Combining G-AnMBR and AnOMBR may represents some synergy by combining the benefits of both technologies in allowing low fouling propensity, low energy requirement, production of biogas and increasing permeate quality thanks to the high rejection rate of FO membranes, decoupling HRT and SRT and increasing organic matter degradation. In this study, we evaluated the progressive substitution of MF membranes by FO membranes in a granular bioreactor to evaluate the concept of Granular Anaerobic Osmotic MBR (G-AnOMBR). For this purpose, FO modules were manufactured to fit in the reactor design having the same size and shape as MF modules. The continuous substitution of MF to FO modules leads us to different hybrid configurations (100% MF, 70% MF, 40–60% MF, 10–20% MF), from which we retrieved information regarding salinity increase, membrane fouling, organic matter removal, hydraulic retention time, flows and other variables.

## 2. Materials and Methods

### 2.1. Pilot Scale Set-Up and Operating Conditions

**Figure 1:**
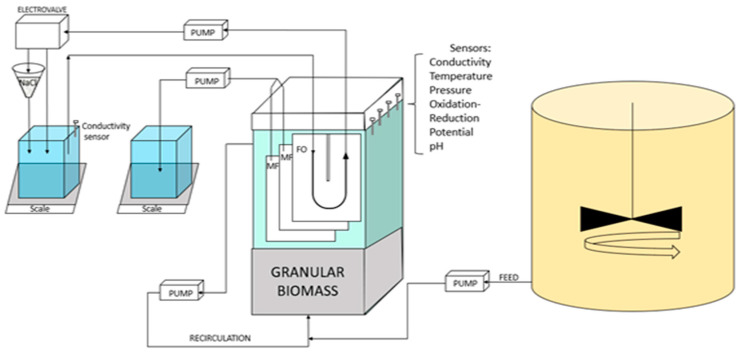
Experimental setup of the FO-G-AnMBR pilot.

The pilot scale described in Figure 1 features a rectangular parallelepiped reactor (282 × 100 × 900 mm) with a working volume of 17 L. Up to 3 flat sheet membrane modules (MF and/or FO) with a filtration surface area of 0.1 m^2^ each were placed in the reactor. Kubota MF modules 203 were used as MF plates. FO modules were of home-made build based on PVC support and using a new generation of thin film composite (TFC) commercially available FO membranes obtained from Toray Industries (Seoul, South Korea) as used in our former study [21]. The FO modules featured a U-shape draw channel design (as commonly found in spiral-wound FO modules with draw channel spacers containing a 1.2-mm-thickness diamond-type polypropylene mesh spacer composed of two levels of filaments to promote turbulence [21]). Characteristics of the TFC FO membrane are as follows. Permeability to water (at 20 °C): 8.9 ± 0.14 L m^−2^ h^−1^ bar^−1^; Permeability to NaCl: 5.68 ± 0.14 L m^−2^ h^−1^; Structural parameter: 466 × 10^−6^ m [22]).

FO modules were tested before use in the G-AnMBR reactor to check both for integrity and performances to define the draw solution (DS) concentration required using a similar setup to that in our former study [23]. The RSD value of FO modules was measured before the operation of the reactor and was in line with former works/values for similar TFC Toray membrane modules, i.e., 0.6 g.L^−1^ (g NaCl/L of permeated water) [22,24]. Given the specific operating conditions of the G-AnOMBR reactor (no gas sparging, WW as feed solution, expected permeation flux of 5 ± 1 L·m^−2^·h^−1^), DS concentration was defined at 15 g.L^−1^.

MF and FO modules were operated under negative pumping pressure using 323 S peristaltic pumps (Watson-Marlow, UK) without relaxation or gas sparging. MF permeate flux was controlled by the pump velocity. FO Draw solution was pumped from the draw tank, circulated into the FO modules at a flow rate of 0.24 L.min^−1^ and returned to the draw tank. Permeate flows were monitored by the increase of mass in the permeate and draw tanks using a Kern EWJ balance. Unless later on specific specified conditions, the average targeted permeation flux was of 5 ± 1 L·m^−2^·h^−1^.

The reactor was seeded with 87 g TSS/L of already formed anaerobic granular sludge obtained from a paper mill factory (Laveyron, France) with a volatile fraction of 57%. The hydraulic retention time (HRT) was set at 10 h with the aim to achieve an optimal organic matter removal of 90% [11]. The reactor was fed with synthetic wastewater (Table 1, COD/N/P ratio: 100/5/1) that was prepared and stored in a 175 L stirred metallic tank cooled at 5 °C. The feed COD concentration was of 500 mg.L^−1^. All experiments were conducted in the reactor volume at a temperature of 25 °C. The biomass level filled up the bottom part of the reactor up until the bottom part of the membrane modules. A recirculation pump set at 40 L.h^−1^ was used to assure a good contact in-between the WW and the biomass and a slight microbial granules fluidization.

The pilot was fully monitored and controlled by a homemade Arduino system. Oxidation-reduction potential, conductivity, temperature, and pH sensors were placed in the G-AnMBR reactor supernatant. Transmembrane pressure was measured using a pressure sensor in the MF permeate line. All sensors were Arduino compatible and purchased from DF Robot (China). A level sensor was placed in the reactor to maintain constant reactor volume at 17 L; whenever the reactor volume decreased, WW was pumped automatically into the reactor. Draw salinity was set at 15 g.L^−1^, i.e., conductivity of 23 mS.cm^−1^, and was adjusted based on a conductivity sensor that was placed in the draw solution tank and which controlled an electro valve. If conductivity was below 23 mS.cm^−1^, the electro valve sent the draw solution coming back from the modules to a funnel filled with sea salts placed over the draw solution to adjust the conductivity. All data were registered in an SD memory card connected to the Arduino system every 15 min.

### 2.2. Operation of the G-AnMBR Reactor

After setting-up the optimized conditions, the G-ANMBR reactor was operated during 10 days in configuration 100% MF (using 3 MF membrane modules). Then, one MF module was substituted by an FO module and maintained during at least 10 days in this new operating mode. Step by step, MF modules were substituted by FO modules. Thus, the reactor was successively operated with various MF/FO extraction ratios which were calculated based on actual permeation flux during each tested configuration:

- 3 MF modules: 100% MF
- 2 MF modules/1 FO module: 70% MF/30% FO
- 1 MF module/2 FO Modules: 40–60% MF/60–40% FO
- 1 MF module/2 FO Modules: 10–20% MF/80–90% FO (MF operated at low permeation flux)

MF was operated at constant flux and therefore fouling occurrence was assessed through TMP increase. MF membranes were cleaned before changing the NF/FO ratio as well as whenever the TMP increased above 300 mbar. Cleaning consisted in (1) flushing of the fouling layer using 1 L of DI water and (2) chemical cleaning by immersion in sodium hypochlorite at 20 mg.L^−1^ during 1 h. FO membrane fouling was assessed through permeation flux reduction; once 30% flux was lost, membranes were cleaned by (1) flushing with 0.5 L of DI water followed by osmotic backwashing during 1 hour using DI water and 70 g.L^−1^ sea salts solution. Membrane permeability and integrity tests were performed after cleaning protocols and demonstrated full recovery of initial performance. For both FO and MF membranes, biofilms removed with the 0.5 L DI flushing of each cleaning were kept for further characterization (3DEEM, protein content, polysaccharide contents, total solids and volatile solids).

### 2.3. Chemical Oxygen Demand

During each step, total chemical oxygen demand (COD) analyses were realized every 2 days to check the organic matter removal, taking samples from the feed, reactor and permeate and using Lovibond kits (COD Vario Tube Test 0–1500 mg/l and 0–150 mg/L) and spectrophotometer (Photometer-System MD100).

With COD being fully rejected by the FO membrane, the remaining COD could only be released via the MF permeate. In order to take this into account, the actual COD removal (%COD removal) of the system was calculated, based on Feed flowrate (Q_Feed_), MF permeate flowrate (Q_MFP_) and COD content of the feed (COD_Feed_) and MF permeate (COD_MFP_) as in Equation (1):

(1) $$\%\mathrm{COD}\;\mathrm{removal}=\frac{{\mathrm{COD}}_{\mathrm{MFP}\times }Q_{\mathrm{MFP}}}{{\mathrm{COD}}_{\mathrm{Feed}\times }Q_{\mathrm{Feed}}}$$

### 2.4. Ion Analysis

Feed, reactor, MF permeate and draw solution samples were taken for each MF/FO ratio in order to estimate (1) potential salt concentration in the reactor and in MF permeate and (2) salts passage between the reactor and the draw solution. The concentration of soluble cations (ammonium (NH_4_^+^), sodium (Na^+^), potassium (K^+^), magnesium (Mg^2+^), and calcium (Ca^2+^)) as well as the concentration of anions (nitrate (NO_3_^−^), chloride (Cl^−^), sulfate (SO_4_^2−^) and phosphate (PO_4_^3−^), were determined using ion chromatography (Method 4110 B, IC5000, Dionex, USA), after filtering samples with 0.2 µm nylon filters. Theoretical (individual) ions concentration (Rt, in mg.L^−1^) was calculated assuming perfect salt rejection by the FO membrane and to estimate the theoretical salt concentration based on the feed ions concentration (I_Feed_, in mg.L^−1^), feed flowrate (Q_Feed_) and their release through the MF permeate (Q_MFP_):

(2) $$\mathrm{Rt}=\frac{I_{\mathrm{Feed}}\times Q_{\mathrm{Feed}}}{Q_{\mathrm{MFP}}}$$

Then, this theoretical concentration was compared with the actual concentration in the reactor (Rt) to estimate potential salts passage through the FO membranes (from the reactor to DS and DS to the reactor). Samples from the DS were also analysed and the composition of the DS was compared with its initial composition, taken from a control DS sample in order to calculate increase of those ions in the DS (ΔDS).

Using Equation (2), overall salinity increase was calculated. Theoretical conductivity increase based on feed conductivity concentration was compared to actual salinity increase to estimate the fraction of the salinity increase due to reverse salt diffusion (RSD) for several data points.

### 2.5. Biomass and Biofilm Analysis

The biomass concentration in the reactor and the biofilm quantity of Total solids (TS) and Volatile solids (VS) were measured according to standard methods [25]. The dissolved organic matter in the developed biofilm was analyzed by three-dimensional excitation emission matrix (3DEEM). Samples were collected from the physical cleaning and pre-filtered at 0.45 µm. 3DEEM were obtained using a Perkin-Elmer FL6500 spectrometer (USA) following methods from [11,23]. In addition, protein (PN) and polysaccharide (PS) contents were measured through Lowry and Dubois methods, respectively, to follow any modification or release of these organic compounds during the experiments, following methods described in [23].

## 3. Results

Overall, the G-AnMBR with MF and FO was operated for more than 50 days and with 4 successive steps corresponding to different MF/FO extraction ratios, i.e., 100% MF, 70% MF, 40–60% MF, and 10–20% MF. Hereafter we discuss how this ratio impacts organic matter degradation, salinity and fouling behaviour.

### 3.1. Organic Matter Degradation

**Figure 2:**
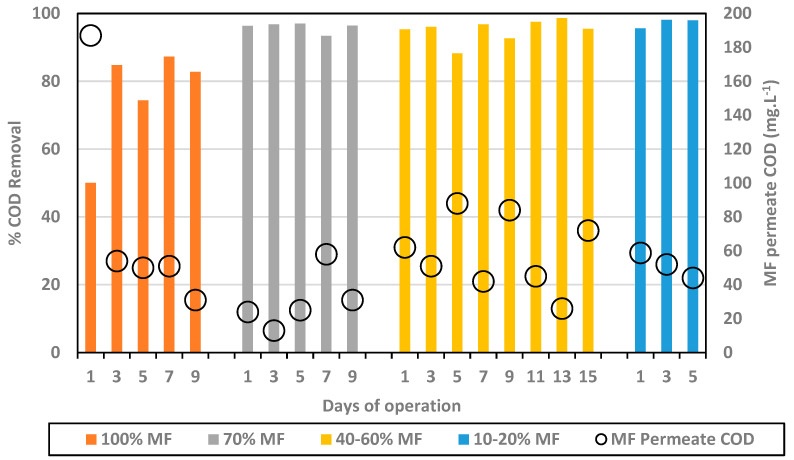
% COD removal (bars) and MF permeate COD concentration (black circles) for every MF/FO ratio.

The % COD removal and COD MF permeate concentration are presented in Figure 2.

In the initial phase, with 100% MF and 0% FO, COD concentration in the MF permeate decreased down to 31 mg.L^−1^ and an average removal of 82.3%. Initial lower performance (first data point) may be attributed to the acclimation of the biological system. The substitution of MF modules by FO ones into the reactor led to an improvement of the overall COD removal well above 90%, leading to average values of 95.9% for 70% MF; 95.0% for 40–60% MF; and 97.2% for 10–20% MF. As such, this indicates first that the integration of FO modules did not decrease the efficiency of the biological process. Moreover, FO modules integration generated only water extraction, due its non-porous composition, leaving the COD fraction within the reactor. In fact, it could be observed that the COD fraction in the reactor was similar to higher than in 100% MF configuration (especially when operating with 40–60% MF). The COD content did not decrease in the MF permeate, remaining below 100 mg.L^−1^ for all tested conditions and close to 50 mg.L^−1^ in most cases.

Higher COD removal obtained when operating with FO membranes can be explained by the higher retention time of the COD fraction in the reactor, the COD rich fraction being extracted only through the MF permeate. As already observed in other studies, FO integration allows for a full dissociation of HRT and SRT, increasing the overall COD fraction degradation. Importantly, the higher efficiency of the system did not lead to a lower COD concentration in the MF fraction but relies on the fact that the MF permeate flow decreased significantly. Based on COD removal efficiency, the most attractive configuration is the 10/20% MF one which led to very high removal efficiency (95–98%) with MF permeate production limited to 10–20% of the treated volume while 80/90% of the inlet feed water could be recycled through the FO system for high quality water production.

### 3.2. Salinity Increase and Salts Passage through FO Membranes

The integration of FO membranes in the G-AnMBR led to a salinity increase in the reactor. Conductivity increase was monitored during the study. Initial conductivity operating with 100% MF modules remained around 1.25 mS/cm and conductivity increased successively up to 2.6, 6.5 and 9 mS.cm^−1^ when increasing the FO extraction rate (and consequently decreasing the MF% to 70%, 40–60% and 10–20% MF, respectively). One of the effects of a conductivity increase is the loss of the osmotic potential, which leads to a lower FO permeation flux and therefore affects the expected MF/FO extracting ratio. Stabilisation of the system was in general observed within 48 h leading to a constant conductivity in the reactor.

**Figure 3:**
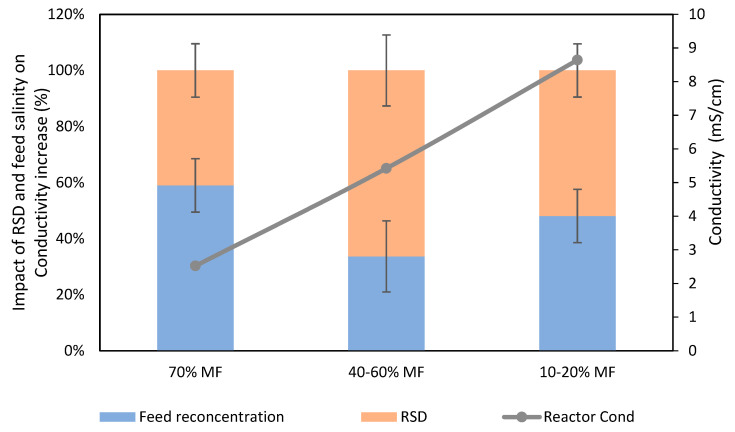
Respective impact of RSD and feed salts concentration on conductivity increase in the G-AnOMBR reactor for each MF/FO ratio.

Salinity increase is the consequence of high feed solution salt rejection by FO membranes leading to salt accumulation in the reactor (as already observed for COD) on the one hand and, on the other hand, RSD from the FO draw solution due to its imperfect salt rejection [26,27]. Based on conductivity measurement, a first assessment was performed to estimate which of those two phenomena was mostly responsible of salinity increase. Theoretical conductivity increase based on feed conductivity concentration was compared to actual salinity increase to estimate the fraction of salinity increased due to RSD for several data points (Figure 3).

It was observed that RSD (NaCl migration from the DS to the reactor) played a significant role in the reactor salinity increase, being already responsible for about 40% of the increase of conductivity when operating with 30% FO extraction and becoming the dominating phenomenon when the % FO extraction increased up to 60%. Such results indicate that selectivity of the FO membrane is a critical aspect to mitigate salinity increase in FO/MBR hybrid processes, which has already been pointed out as a limiting factor for the implementation of such systems. Developing membranes with higher selectivity and the use of draw solution with lower diffusivity or easily biodegradable organic based draw solutions may help to mitigate this effect. Still, even with solving this issue, salinity increase will remain in the FO based bioreactor process and the use of MF/UF membrane as salt purge is most likely necessary.

**Figure 4:**
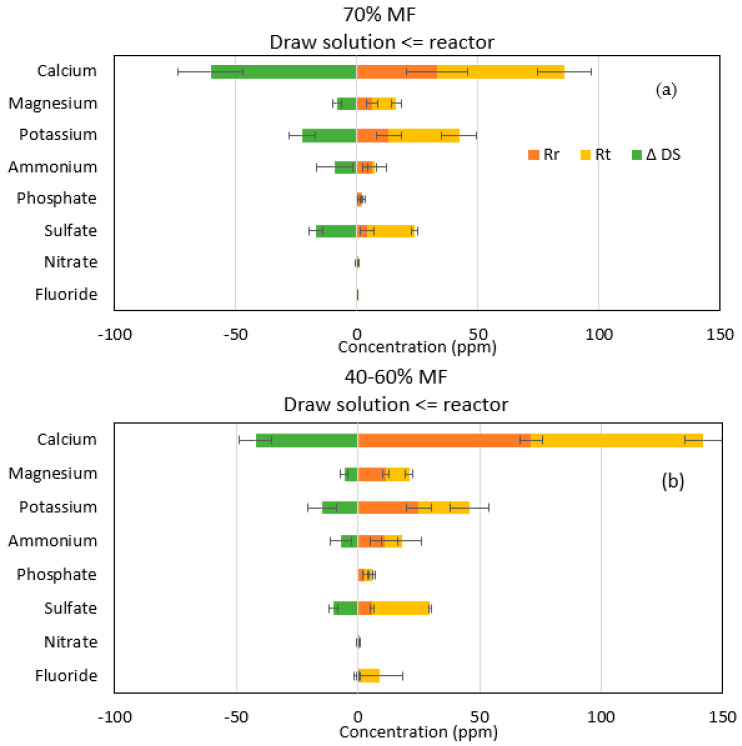
Representation of ion transfer during (**a**) 70% MF and (**b**) 40–60% MF steps. Rr (orange), Rt (yellow) and ΔDS (green) represent the measured concentration, the theoretical concentration in the reactor and the increase of concentration in the DS, respectively.

Ionic chromatography analyses confirmed that sodium and chloride passage were the main ions encountered in the reactor when operating with FO membranes. However, higher migration of sodium than chloride was observed indicating that more complex salt diffusion than just strictly NaCl migration occurred through the FO membrane. Therefore, more in-depth analysis was performed on all other major ions initially present in the feed solution and their theoretical concentration (R_t_) when assuming perfect rejection by the FO membrane. Rt was compared to the actual concentration of those ions in the reactor Rr; the increase of those ions in the DS (ΔDS) was also calculated based on its initial composition, taken from a control DS sample (Figure 4).

For the majority of ions R_r_ was below R_t,,_ indicating that those ions have passed through the membrane to the DS; confirmed by the increase in ΔDS (Figure 3). This phenomenon is known as forward salt diffusion (FSD) [28]. Apart from sodium and chloride, calcium is the main ion transferred to the draw solution. In general, it could be also observed that more cations (calcium, potassium, magnesium) diffused through the FO membrane than anions (ammonium and sulfate). Lower diffusion of anions than cations could be explained by the fact that the system achieves its electroneutrality; higher FSD of cations compensating higher RSD of sodium versus chloride [29,30].

The faster diffusion of cations through the FO TFC membranes can also be explained by electrostatic interactions between ions and the membrane surface. The TFC membrane surface features more negatively charged carboxyl groups, which could serve as a fixed ionic group, therefore conferring to the membrane a cation exchange feature [31]. Other studies have demonstrated that negatively charged membranes had a better rejection of negatively charged compounds while positively charged ones were more poorly rejected [32,33], explaining that negatively charged ions from the FS are more rejected while cations have more affinity.

This study confirmed also that even if monovalent ions diffuse preferentially through the FO membranes, significant divalent ion migrations were also observed due to electrostatic interactions and that simple conductivity analysis is not sufficient to model all ionic interactions in complex FO systems. Phosphate, ammonium and nitrate ions may also have migrated through the membranes, although to a lower level and furthermore loss of those ions may also be partly attributed to biological degradation, use or transformation.

### 3.3. Fouling

Membrane fouling is one of the main problems that affect the performance of the MBR system, leading to a lower volume permeated, increased required operating pressure and operating costs associated with cleaning, and ultimately to reducing membrane life expectancy. Strict comparison of fouling of FO and MF membrane is challenging since they rely on different driving forces (osmotic and hydraulic pressure, respectively). MF membranes are typically operated at constant flux, with fouling occurrence leading to an increase of the filtration resistance, assessed through TMP increase. The FO system was operated at constant draw solution, and fouling occurrence was evaluated through losses in permeation flux.

**Figure 5:**
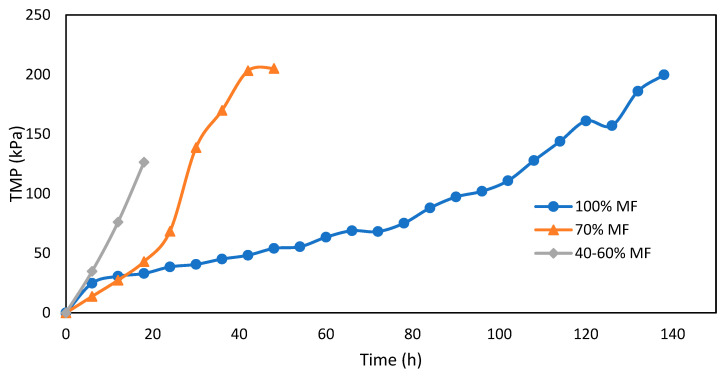
TMP increase during MF operation with various FO/MF extraction ratios and operation at constant flux (5 L.m^−2^.h^−1^).

Fouling of MF membranes assessed through TMP measurements for various MF/FO ratios is presented in Figure 5. The increase of TMP is higher with every FO integration step; it took around 120 h to reach 120 kPa of TMP under normal conditions (100% MF), but decreased to 40 h and less than 20 h for the 70% MF and 40–60% MF, respectively. There are no results for the 10–20% MF due to some issue with the pressure sensor but very quick permeate flux reduction was observed requiring several pump velocity increases to maintain constant permeation flux and indicating a very severe fouling propensity.

**Figure 6:**
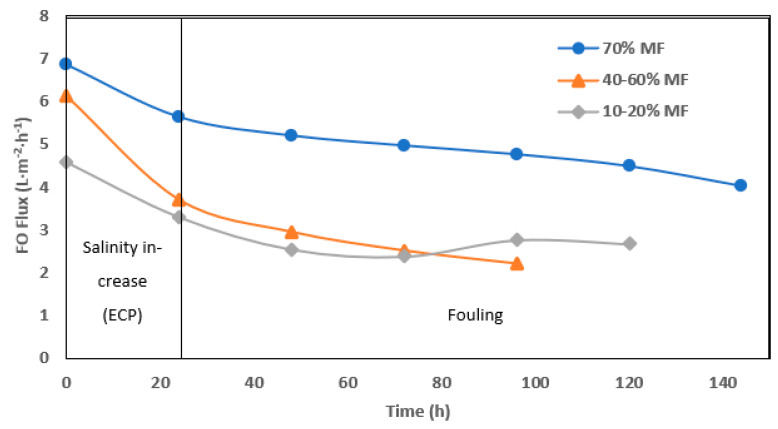
Daily average of FO permeation flux with 70%, 40–60%, and 10–20% of MF.

The fouling rate in FO operation was evaluated through permeation flux (Figure 6) but interpretation remain more complicated due to interconnected effects (flux, fouling and external concentration polarization). Higher initial and average operation flux were monitored when operating with 70% MF ratio. In all cases, a significant drop of permeation was observed after 1 day of operation. Such an effect is most likely due to the increase of salinity observed in the reactor every time the system was shifted toward higher FO rate operation. Conductivity increase in the reactor not only decreased the apparent osmotic pressure gradient but also led to external concentration polarization (ECP) at the membrane surface, further decreasing the osmotic pressure efficiency. Moreover, operation without gas sparging and with low recirculation rate could not allow for ECP mitigation as observed in further study [21,24]. At 70% MF rate, the objective of 5± L.m^−2^.h^−1^ permeation could be achieved and maintained during 7 days. At a lower MF/FO operation rate, initial flux decreased below 4 and 3 L.m^−2^.h^−1^ after the first day and during the 5 days of operation, respectively.

**Figure 7:**
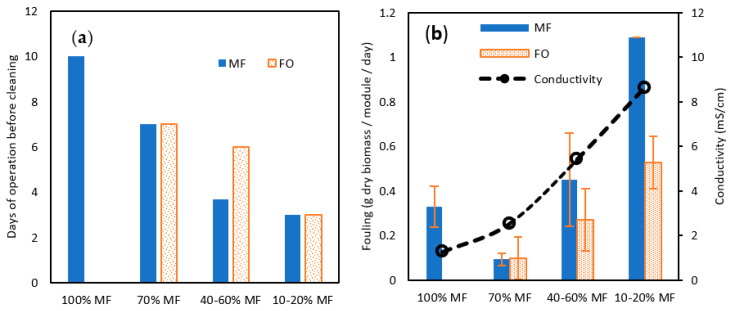
(**a**) Days of operation before cleaning and (**b**) total solid (TS) fouling rate (in g TS/module/day) attached to the membrane surface for MF and FO modules for various MF/FO operating ratios.

Both TMP for MF and permeation flux for FO confirmed the more complicated operation of membrane systems when operating with a higher rate of FO membrane. This conclusion is reinforced by the membrane cleaning frequency, which was reduced from 10 days to 7 days, 4–6 days, and 3 days for 100, 70%, 40–60% ad 10–20% MF ratio steps (Figure 7a). Interestingly, in the 40–60% ratio, FO fouling appeared to be less penalizing and MF and FO modules could be operated for a longer time. To get further confirmation and a fair comparison in-between MF and FO fouling, biofilm samples were collected and dry solids weighed after each cleaning and for each MF/FO ratio (Figure 7b).

At 70% MF operation, the collected amount of biofilm was lower than at 100%, contrasting with former observations regarding TMP increase and operation time; overall, this confirmed 70% MF as an acceptable operating condition. At a higher FO ratio, increased biomass was collected both on FO and MF membranes confirming the higher fouling propensity. The fouling increase could be induced by the raised salinity in the reactor; it has been demonstrated that higher salinity can promote the release of extra polymeric substances (EPS) as well as other halfway compounds derived from uncompleted degradation, which are normally retained inside the granular biomass, due to the cellular membrane plasmolysis [34]. More rapid TMP increase at 70% MF even if a lower biofilm amount was collected could be hypothesised to be the consequence of the higher proportion of EPS substance in the biofilm. Remarkably also, comparing 40–60 and 10–20% steps the collected amount of biofilm was lower for FO membranes than for MF ones, demonstrating the lower fouling tendency of osmotic membranes as previously observed elsewhere [35].

**Figure 8:**
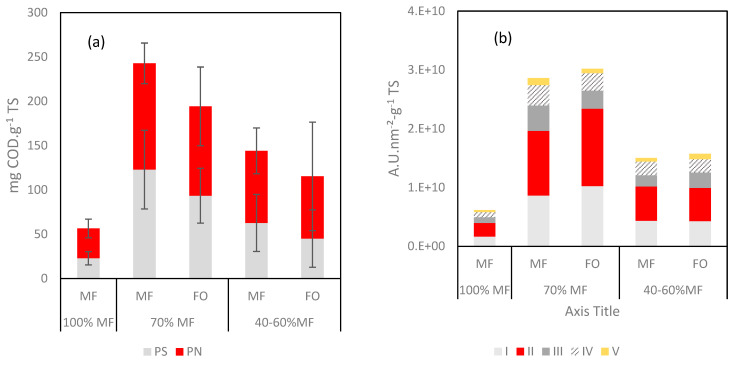
(**a**) Protein (PN) and polysaccharide (PS) contents and (**b**) 3DEEM volume of fluorescence normalized for the fouling layer reported as function of the TS.

Further analyses were performed on the fouling layer with specific quantification through protein (PN) and polysaccharide (PS) contents and 3DEEM fluorescence (Figure 8). With regards to 3DEEM, Regions I + II are associated with protein-like fluorophores, Region III corresponds to fulvic acid-like molecules, Region IV to soluble microbial product (SMP)-like molecules, and Region V corresponds to humic acid-like molecules [23,36].

A high increase of the PN, PS and 3DEEM volume of fluorescence (vs. TS) was observed in all cases once FO was incorporated to the G-AnMBR reactor. When comparing data of 100% MF and 70% MF, it appears clearly that higher fouling rate occurred in the 70% MF configuration despite an overall lower TS deposition rate (Figure 7b). Thus, the higher fouling propensity in 70% can be explained by the major deposition of compounds such as humic acids, EPS, SMP which are comparatively more present in the fouling layer. For the 40–60% configuration, the PS, PN and 3DEEM fractions remain higher than for 100% MF but lower than for 70% MF. The overall behaviour could be hypothesised as the consequence of the initial shock of conductivity following the initial FO integration into the G-AnMBR leading to the release of EPS from the granular biomass. No clear difference was observed regarding the partition of PS/PN and 3DEEM between FO and MF, probably due to the rejection of all these compounds by both membranes.

## 4. Conclusions

In this study, integrated FO/MF G-AnMBR potential has been demonstrated. Introduction of FO membrane modules led to the extraction of high quality permeate through the FO membranes while improving the COD degradation and extraction through the MF permeate. Organic matter removal rate was always above 90% in every MF-FO hybrid configuration operated at ambient temperature. As a main limitation, the high selectivity of FO membranes led to increased conductivity in the reactor which decreased the osmotic driving force and led to a potential cellular plasmolysis and EPS liberation from the granular biomass leading to increased fouling propensity. Operating in fully FO mode does not appear viable due to high salinity increase. MF presence is vital to stabilize reactor salinity generated by FO, with MF actuating as a “salt purge”. Improving FO membrane selectivity would mitigate salinity increase and therefore the operation of the MF/FO hybrid at a higher FO ratio. Further work will help to assess FO/MF G-AnMBR hybrid systems optimization with regards to biogas production (as methane) and enhanced nutrient recovery. In addition, the use of biogas as a gas sparging strategy to limit fouling may help to improve system sustainability and long term operation.

## Figures and Tables

**Table 1:** Feed wastewater composition.

Substrate	NaCH_3_COOH	C_6_H_12_O_6_	NH_4_Cl	CaCl_2_, 2H_2_O	MgSO_4_	KCl	KH_2_PO_4_	NaHCO_3_
Concentration (mg.L^−1^)	354	156	64	18	16	30	15	200
